# Therapeutic effect of *Siddhar yogam* on varicose veins - A pilot clinical trial

**DOI:** 10.6026/973206300223343

**Published:** 2026-06-30

**Authors:** Manobala V.R, Mahalakshmi V, Sugasini P, Manivannan P, SivaNandini T.K, Naveena E, Senthilvel G

**Affiliations:** 1 Department of Siddhar Yoga Maruthuvam, National Institute of Siddha, Chennai - 47, (Affiliated to The Tamilnadu Dr.M.G.R. Medical University, Chennai), Ministry of Ayush, Chennai-47, Tamilnadu, India; 2 Department of Sirappu Maruthuvam, National Institute of Siddha, Chennai - 47, India; 3 National Institute of Siddha, Chennai - 47, India

**Keywords:** Varicose veins, *Siddhar yogam*therapy, Siddha medicine, Aberdeen Varicose Vein Questionnaire (AVVQ), Yoga therapy

## Abstract

Varicose veins are dilated and twisted subcutaneous veins of the lower limbs and represent one of the most commonly reported conditions. The lack of standardized, non-invasive therapeutic interventions for the management of varicose veins often leads to persistent physical discomfort and reduced quality of life for patients. Therefore, it is of interest to evaluate the effect of *Siddhar yogam* on varicose veins among patients reporting at Ayothidoss Pandithar Hospital, National Institute of Siddha, using Aberdeen Varicose Vein Questionnaire (AVVQ). A single-arm open-label pilot clinical trial was conducted in 30 patients who practiced the prescribed *Siddhar yogam* protocol for 90 days and outcomes were assessed before and after intervention. Thus, we show a significant reduction in symptoms, indicating the potential effectiveness of *Siddhar yogam* in managing varicose veins.

## Background:

Siddha system is the traditional system of medicine that originated in South India. The Siddha system constitutes a comprehensive healthcare paradigm that integrates preventive, promotive, curative, rejuvenative and rehabilitative modalities. Siddha system works through the basic principle of *Vatham* (Air), *Pitham* (Fire) and *Kabham* (Water) humors which are called *uyir thathukkal* (Vital Humors). Derangement *i.e.*, increase/decrease in any one or more of these humors, may cause illness in human health [1, 2]. *Siddhar yogam* is one of the *Kayakarpam* (Rejuvenation science) methods of the Siddha System of Medicine. It is not only a meditation science, but also comprises eight types of Siddhis called *Ashtanga Yogam*, which includes *Iyamam* (Ethical Standards), N*Iyamam* (Self-Discipline), *Asanam* (Yogic Postures), *Pranayamam* (Breath Control), Prathiyakaram (Withdrawal of the Senses), Dharanai (Concentration), *Dhiyanam* (Meditation) and *Samaadhi* (Enlightenment) [3, 4]. *Siddhar yogam* offers a holistic approach to health and wellness. It is a combination of specific yoga postures, breathing techniques and meditation techniques for physical and mental well-being. Varicose veins are a disorder of the venous system marked by subcutaneous dilated, tortuous veins greater than or equal to 3 millimetres, affecting the saphenous vein, its tributaries, or non- saphenous superficial veins in the lower limbs [5]. Varicose veins are a problem for people who stand for longer than four hours a day [6, 7]. Varicose veins may cause cramping pain in the affected legs and movement problems in the lower extremities. The risk factors of varicose veins include prolonged standing, overweight, aging, smoking, heavy weight lifting and pregnancy in females. An increase in straining bowel movement, low fibre intake, genetic weakness in vein walls and menopause can also increase the chances of developing varicose veins [8]. Varicose veins affect 2-75% of the global population. In the Western world, 10-20% of the population is affected. Varicose veins affect 5% of the population in India [9, 10]. The prevalence of varicose veins is higher in women [11]. Varicose veins are not life-threatening, but they have been considered a cosmetic problem that causes a considerable demand on medical care [12, 13]. The CEAP classification is important for diagnosing varicose veins; nevertheless, it lacks utility in directing or stratifying therapeutic interventions. Therapeutic approaches for varicose veins encompass both conservative management and invasive interventions, including modalities such as thermal ablation, endovenous sclerotherapy and surgical procedures [14, 15]. According to T.V. Sambasivam Pillai's dictionary, varicose vein is quoted as "*Narambu Kirandhi*," a condition in which the veins are enlarged or dilated. This Enlargement or dilation is due to sluggishness or impediment to the circulation of the blood through the veins and this sluggishness may depend on debility or many other causes. The veins may be seen cluster in raised knots under the skin in different directions [16]. In the Siddha system of medicine, varicose veins is caused by derangement in *Vatham* (Air) and *Kabham* (Water) humors and some siddhars said that it is also due to derangement in the *Udal thadhus* (Body constituents) such as blood (*Kuruthi*), muscle (Sathai) and fat (Kozhuppu) [17, 18]. In Siddha, varicose veins are referred by various terms, including *Narambu silanthi*, *Naala pudaippu*, *Narambu kiranthi* and *Naala vibatham* [19]. Therefore, it is of interest to report the therapeutic effect of *Siddhar yogam* in the management of varicose veins.

## Materials and Methods:

## Study participants:

An open-label, single-arm, interventional pilot clinical trial was conducted on 30 patients who visited the outpatient department of Siddhar Yoga Maruthuvam at Ayothidoss Pandithar Hospital, National Institute of Siddha, with symptoms of varicose veins. A sample size of 30 was selected as the minimum number of participants required for a pilot clinical trial to assess the feasibility and safety outcomes. Out of 45 patients screened, 30 met the inclusion criteria. The criteria included all genders, aged 30-55 years, with CEAP grades 1 and 2 varicose veins, up to 3 years of duration. The exclusion criteria were patients with a known history of chronic renal failure, cardiac disorders, diabetic ulcers, varicose ulcers, cellulitis, deep vein thrombosis, patients with recent surgical and traumatic history and pregnant and lactating mothers. The study was reviewed by the Institutional Review Board and approved by the Institutional Ethics Committee and Informed consent was obtained from all the study participants.

## Intervention:

The prescribed *asanam*s were advised for the participants. Each *asanam* was performed for 2 minutes and relaxation in between each *asanam* was one minute. Prescribed *Yogasanam* were taught to the participants on the first visit and advised to follow for 90 days, also during each visit of the hospital. Participants were advised to practice *yogasanam* on an empty stomach after evacuation of urine and faeces every morning and evening.

## Yogam Protocol:

Total timing: 35 minutes

Relaxation techniques 5-10 minutes.

[1] *Asanam*: 21 minutes

[2] *Uttanapadasanam* (Raised leg pose)

[3] *Ardha halasanam* (Half plough pose)

[4] *Vibaritha karani* (Inverted pose)

[5] *Pada sanchalasanam* (cycling pose)

[6] *Salabasanam* (Locust pose)

[7] *Paschimottanasanam* (Forward stretch)

[8] *Shavasanam* (Corpse pose)

[9] *Pranayamam*: *Thirumoolar Pranayamam* - 5 minutes

## Conduct of study:

30 participants were selected as per the inclusion and exclusion criteria. Selected *Yogasanam* was taught and advised to practice for given time. The enrolled participants visited the hospital once in 7 days. The Aberdeen Varicose Vein Questionnaire (AVVQ) score was calculated before and after treatment Figure 1 (see PDF).

## CTRI:

The clinical trial was registered with Clinical Trial Registry of India (CTRI/2024/06/068899).

## IEC:

The clinical trial was carried out according to the defined protocol, following the approval of the Institutional Ethical Committee (IEC) 20/04/2024;NIS/26/IEC/2024/M.P/32

## Outcome measures:

Demographic details and Baseline characteristics of the participants such as Age, Gender, Occupation and Family history, were collected. AVVQ Score was used to assess the improvement before and after 90 days of intervention [23]. Siddha diagnostic parameters such as *Envagai thervu *(Eight-fold examination), *Mukuttram*, *Udal thathukkal* (Body Constituents), were assessed before and after the intervention.

## Statistical analysis:

The mean and standard deviation of the AVVQ scores of the participants before and after the treatment were calculated. The AVVQ scores before and after the intervention period were analysed by the Wilcoxon signed-rank test.

## Results:

A total of 45 patients with varicose veins were screened from the OPD of Ayothidoss Pandithar Hospital, of which 15 patients were excluded from the study for not meeting the selection criteria. The remaining 30 patients were included in the study. Out of the 30 participants recruited in the study, there were 10 Male (33.3%) and 20 Female (66.7%). 50 % (N = 15) of the study population were between the age of 30 - 40 years. The remaining 30 % (N=9) of the study population belonged to the age group of 40 - 50 years and 20 % (N = 6) of the study population were between 50 - 55 years. Only 10 % (N = 3) of the study population has a positive family history of varicose veins, while 90 % (N = 27) of the study population has no family history of varicose veins. According to the occupational status, majority of the study population was comprised of housewives (40%), followed by Shopkeepers (23.3%), Software engineers (13.3%), Tea shop vendors (6.6%), Security (6.6%), Teacher (6.6%) and the least being participants with business profiles (3.3%). Over 85% of the study population reported a history of prolonged standing. *Naadi* assessment of the study participants revealed that the majority of study participants, about 46.6 % had *Vathapitha naadi*, followed by *Pithavatha naadi* (33.33 %), *Pithakabha naadi* (13.33 %) and *Vathakabha naadi* (6.66 %). *Abaanan*, *Viyanan* and *Samaanan* are the primary *vaayus* affected. *Sathaga Pitham* and *Pirasaga Pitham* are the affected subtypes of *Pitham* in the study population. No affected subtypes of *Kabha kutram* is observed in the study population. Of the *Envagai thervu* (Eight-fold examination) in *Siddha*, *Sparisam*, *niram* and *malam* are affected in the study population in various proportions. *Udal thathukkal* (Body constituents) assessment revealed the deranged *Saaram*, *Senneer* and *Oon Thathus*. The Mean ± SD of the AVVQ score reduced from 16.2 ± 1.8 before the treatment to 12.0 ± 2.9 after the treatment. The Mean scores of the 30 participants before and after the treatment were tested significant difference using the Wilcoxon signed rank test. It showed a significant reduction in mean scores (p < 0.05) before and after the treatment, which are detailed in Table 1.

## Discussion:

In this single-arm, non-randomised clinical trial for varicose vein treated with *Siddhar yogam* regimen for 90 days in 30 participants, resulted in reduction of clinical symptoms and improvement of quality of life as evident from the decrease in AVVQ Scores. The assessment of *Mukuttram* revealed the derangement of the *Vaayus* (*Abaanan*, *Viyaanan and Samaanan*) and *Pitham* (*Sathagam and Prasagam*). According to *Envagai thervu* assessment, *Sparisam*, *Niram* and *Malam* are affected. The assessment of *Udal thathukkal*, revealed the affected *Saaram*, *Senneer* and *Oon thathukkal*. The assessment of *Mukuttram*, *Envagai thervu* and *Udal thathukkal* before and after the treatment showed a reduction in the affected parameters among the study population to various extents. *Narambu kiranthi* is mainly caused by the stagnation of blood in blood vessels, especially the veins, either due to obstruction to the flow of blood or any other cause. The stagnation of blood in the vein leads to an increase in *Vatham* humour which produces dryness inside the stagnated vessels [17, 18]. *Vatham*, having the qualities of *ilagu* leads to the dilation of vessels and appearance of knotty, circular structures protruding on the skin [20]. Among the 10 *Vaayus*, *Abaanan Vaayu* being the downward force, its derangement causes the stagnation of blood in the veins which then affects the *Vaayu Viyaanan*, which is involved in the exchange of fluids between the Vessels and extracellular space and the *Vaayu Samanan*, which maintains the homeostasis between various *vaayus* [20]. The affected *vaayus* then affects the other humors leading to the disease. There are numerous studies advocating the efficacy of yoga in treating varicose veins. Chauhan *et al.* studied the combined effect of yoga and naturopathy in patients with uncomplicated varicose veins through a prospective randomized controlled trial. Outcomes were assessed using AVVQ (p<0.01) and VAHS scores (p<0.05), which showed statistically significant differences between the experimental and control groups. The study also evaluated anthropometric measurements, BMI, blood pressure and Hs-CRP levels. It was observed that Hs-CRP levels decreased in the experimental group compared to the control group [21]. Yamuna *et al.* studied the effect of yoga on chronic venous insufficiency in industrial workers through a randomized controlled trial, revealing a reduction in plasma homocysteine levels between the experimental and control groups, which was the primary outcome. They also observed a significant reduction in the Venous Clinical Severity Score, CVI questionnaire and Chalder fatigue scale between the groups after 12 weeks of treatment [22]. The aasanas *Uttanapadasanam*, *Vibarithakarani*, *Ardha halasanam*, *Pada sanchalasanam*, *Salabasanam* and *Paschimottasanam* regulates the intra-abdominal pressure thereby reducing the stagnation of blood, strengthening and toning the abdominal and lower limb muscles, leading to reduced vein pressure. These asanas enhances the venous return and reduces pressure in the valves of the veins by promoting blood flow from the lower limbs to the heart. Inverted and dynamic poses stimulate the calf muscle pump, improve circulation, reduce venous pooling and strengthen leg muscles, thereby alleviating symptoms of varicose veins. *Shavasanam* promotes general relaxation of the body muscles, improving circulation. The main limitation of the study is that, the study being a single-arm, non-randomised clinical trial, the efficacy of the treatment regimen cannot be established due to the absence of a comparison group. Long-term follow-up of the patient post-treatment was not done; hence, the recurrence of the disease after treatment withdrawal cannot be determined. Only the AVVQ score was used as an outcome variable, which is a limitation of the study. Various tools like VAHS Score, Hs-CRP and Plasma homocysteine levels may be used as Outcome variables along with AVVQ score before and after the treatment. Another limitation of the study is that the study population does not involve severe varicose vein patients to ascertain the full potential of *Siddhar yogam* therapy in varicose veins.

## Conclusion:

*Siddhar yogam* has a positive impact on the management of varicose veins. The 90-day *Siddhar yogam* protocol resulted in a noticeable reduction in the clinical symptoms of varicose veins and improvement in overall quality of life. Thus, regular practice of *Siddhar yogam* can enhance venous circulation and reduce discomfort. Further large-scale randomized controlled trials will be helpful to establish standardized *Siddhar yogam* protocols for clinical practice.

## Sources of funding:

None

## Figures and Tables

**Table 1:** Wilcoxon signed rank test

**Variable**	**Ranks**	**N**	**Mean rank**	**Test statistics**	**P value**
	Negative ranks	25	6.24		
AVVQ (Post-Pre)	Positive ranks	0	0		
	Tie	0		-4.372	0.0001
	Total	30			
